# Genome assembly of the foot-flagging frog, *Staurois parvus*: a resource for understanding mechanisms of behavior

**DOI:** 10.1093/g3journal/jkad193

**Published:** 2023-08-25

**Authors:** Mika A Holtz, Riccardo Racicot, Doris Preininger, Adam M M Stuckert, Lisa A Mangiamele

**Affiliations:** Department of Biological Sciences, Smith College, Northampton, MA 01053, USA; Department of Biological Sciences, Smith College, Northampton, MA 01053, USA; Vienna Zoo, 1130 Vienna, Austria; Department of Evolutionary Biology, University of Vienna, 1030 Vienna, Austria; Department of Biology & Biochemistry, University of Houston, Houston, TX 77204, USA; Department of Biological Sciences, Smith College, Northampton, MA 01053, USA

**Keywords:** *Staurois parvus*, foot-flagging frog, genome, RNAseq, communication behavior

## Abstract

Elaborate and skilled movements of the body have been selected in a variety of species as courtship and rivalry signals. One roadblock in studying these behaviors has been a lack of resources for understanding how they evolved at the genetic level. The Bornean rock frog (*Staurois parvus*) is an ideal species in which to address this issue. Males wave their hindlimbs in a “foot-flagging” display when competing for mates. The evolution of foot flagging in *S. parvus* and other species is accompanied by increases in the expression of the androgen receptor gene within its neuromuscular system, but it remains unclear what genetic or transcriptional changes are associated with this behavioral phenotype. We have now assembled the genome of *S. parvus*, resulting in 3.98 Gbp of 22,402 contigs with an N50 of 611,229 bp. The genome will be a resource for finding genes related to the physiology underlying foot flagging and to adaptations of the neuromuscular system. As a first application of the genome, we also began work in comparative genomics and differential gene expression analysis. We show that the androgen receptor is diverged from other anuran species, and we identify unique expression patterns of genes in the spinal cord and leg muscle that are important for axial patterning, cell specification and morphology, or muscle contraction. This genome will continue to be an important tool for future -omics studies to understand the evolution of elaborate signaling behaviors in this and potentially related species.

## Teaser

*Staurois parvus* is a frog species in which males use a “foot flagging” display to rival each other and attract mates. Holtz et al. present its newly sequenced genome as a resource for coming studies of the genetic underpinnings of foot-flagging behavior. Additionally, the authors observed significant divergence of the androgen receptor sequence from other species and conducted differential expression analysis to identify genes in the hind limb neuromuscular pathway that may be related to the behavior.

## Introduction

Sexual selection has resulted in a diversity of elaborate and physically impressive behaviors that have evolved in males to compete with rivals and attract mates (Byers *et al*. 2010; Barske *et al*. 2011). In many cases, these behaviors evolve by co-opting existing physical capabilities, but in other cases this requires the evolution of novel motor skills (reviewed in Fuxjager *et al*. 2022; Goller 2022). The emergence of such motor skills is presumably accompanied by modifications to neural circuits and their connected muscles, which control how the body and limbs are moved to enable impressive feats of acrobatics, coordination, and/or rapid movements (Fuxjager *et al*. 2022; Schwark *et al*. 2022). Yet, little is known about these neuromotor adaptations and their underlying genetic mechanisms (Fuxjager *et al*. 2016; Pease *et al*. 2022).

**Fig. 1.**
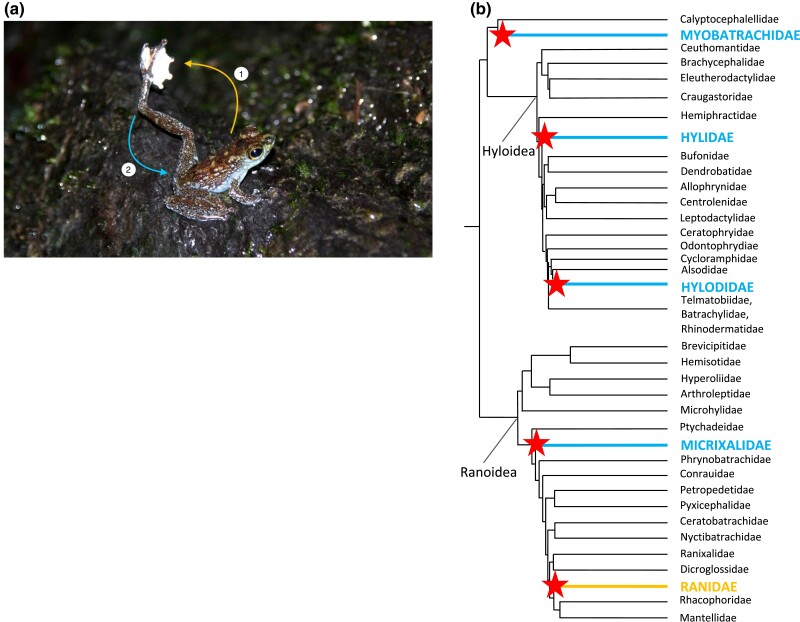
a) A *Staurois parvus* male foot flagging. The hind foot is raised above the head in a slow, arcing motion (1), while displaying bright-white foot webbing, and then quickly retracted back into the body (2). Photo credit: Doris Preininger. b) Family-level phylogeny showing independent evolution of foot-flagging behavior (denoted with stars) in diverse anuran clades. *S. parvus* belongs to the family Ranidae. Phylogeny pruned from Pyron and Wiens 2011.

To address this issue, we study the Bornean rock frog (*Staurois parvus*), which has a novel form of hind limb display called “foot flagging” (Fig. 1a; Supplementary Video 1) that is used primarily in agonistic encounters with other males at breeding sites. Foot flagging has convergently evolved 5 times in the anuran lineage (Fig. 1b), arising in roughly 2 dozen species (Hödl and Amézquita 2001). The independent evolution of similar displays across the anuran phylogeny provides an opportunity to test whether common neuromuscular mechanisms have evolved in foot-flagging species or whether there are many mechanistic solutions that produce similar motor behaviors. For example, in *S. parvus*, foot flagging is an androgen hormone-mediated behavior (Mangiamele *et al*. 2016; Smith *et al*. 2021) and the evolution of foot flagging in multiple anuran lineages is associated with dramatic changes in tissue sensitivity to androgens. Compared with frogs that cannot foot flag, *S. parvus* and other foot-flagging species have much higher androgen receptor (AR) expression in their hind limb muscles (Mangiamele *et al*. 2016; Anderson *et al*. 2021). These findings give us some insight into how novel motor behaviors could evolve through changes in neuromuscular gene expression. However, in order to make further progress, we are in need of a resource that enables us to uncover sources of genetic variation between different individuals and species and to probe the functional consequences of such variation (Miller *et al*. 2019; Gallant and O’Connell 2020).

Next-generation sequencing has made it possible to access genomic information in “non-model” species and to identify suites of genes that are expressed in specific neuromuscular tissues. Here, we introduce the genome of the foot-flagging frog, *S. parvus*. Using PacBio CLR sequencing, we produced a 3.98-Gbp genome assembly with a total of 22,402 contigs (contig N50 of 611,229 base pairs), representing 91.4% of conserved vertebrate genes. Interestingly, we have found significant divergence in AR in the lineage that led to *S. parvus.* We also present an RNA sequencing analysis that identifies patterns of gene expression within relevant neuromuscular pathways. The genome will be a critical resource for future comparative work that will help identify other genes that could be relevant to the evolution of foot-flagging behavior.

## Methods

### IACUC statement

Adult male *S. parvus* frogs were collected from a captive colony at the Vienna Zoo in Vienna, Austria, which was derived from wild frogs collected in Ulu Temburong National Park, Brunei Darussalam in 2010. Animals were maintained, and our tissue collection protocol was approved, by the appropriate Institutional and Animal Care and Use Committees at Smith College (ASAF#17R-LM-290), the University of Vienna (Protocol #2014-013), and the Vienna Zoo.

### Genomic DNA extraction and sequencing

We generated genomic DNA from a single adult male frog using whole-body fresh tissue. We completely removed the skin and dissected out and discarded intestinal tissue. We rinsed the tissue with 1× sterile PBS and ground it to a fine powder in a pre-chilled mortar and pestle. The ground tissue was transferred to a tube containing 5 mL of lysis buffer consisting of 1% SDS, 100 mM NaCl, 10 mM Tris HCl pH 8.0, 10 mM EDTA, 40 mM DTT, and proteinase K. Lysis was performed overnight at 55°C on a rotating mixer. Lysate was spun down at 12,000 × *g* for 10 minutes at room temperature, and the supernatant was transferred to a new tube. We added 15 μL of RNase A to the lysate and incubated the sample for 30 minutes at room temperature. We added an equal volume of phenol chloroform isoamyl 25:24:1 to the sample and gently rocked it back and forth for 1 minute and then centrifuged at 12,000 × *g* for 10 minutes at room temperature. We transferred the upper phase to a new tube and an equal volume of chloroform isoamyl 24:1 to the sample which was then gently rocked back and forth for 1 minute and centrifuged at 12,000 × *g* for 10 minutes at room temperature. The upper phase was transferred to another new tube, and 2× volume of absolute ethanol and 0.1 volume of 3M sodium acetate were added. DNA was precipitated for 2 hours at room temperature and then centrifuged at 12,000 × *g* for 30 minutes at 4°C to turn the gDNA into pellets. We removed the ethanol and washed the pellet with 1 mL of fresh 70% ethanol. We centrifuged the gDNA at 12,000 × *g* for 5 minutes at 4°C. We then discarded the ethanol and allowed the pellet to dry before resuspending in 200 μL of 1× TE buffer. gDNA was allowed to go into solution at room temperature overnight on a rotating mixer at 150 rpm. We quantified the gDNA using Nanodrop and Qubit.

We sent the gDNA to the Icahn Institute for Data Science and Genomic Technology at Mount Sinai, New York, New York, for sequencing. PacBio SMRTBell libraries were generated and sequenced on a PacBio Sequel II using continuous long read chemistry. Libraries were sequenced to a depth of 57× coverage, assuming a genome size of 3.98 Gbp, with 227 GB of raw data generated across 3 independent runs.

### mRNA extraction and sequencing

To identify patterns of gene expression associated with neuromuscular tissues involved in motor control and foot-flagging behavior, we dissected out the whole brain, spinal cord, and thigh muscles from adult male *S. parvus* and placed fresh tissue in RNAlater (Thermo Fisher) until further processing. In total, we were able to obtain 2 brains, 4 spinal cords, and 5 leg muscles. To extract mRNA, we rinsed the tissue with 1× sterile PBS, added 1 mL of TRIzol (Invitrogen), and completely disrupted the tissue using a homogenizer. We then added 0.2 mL of chloroform. We gently shook the tubes and allowed them to incubate for 10 minutes. We then centrifuged the samples at 12,000 × *g* for 15 minutes at 4°C and transferred the top phase to a new tube and added 500 μL of isopropanol. The RNA then precipitated overnight at −20°C. We pelleted the RNA by centrifugation at 12,000 × *g* for 30 minutes at 4°C. Pellets were washed with 1 mL of fresh 70% ethanol and centrifuged at 12,000 × *g* for 5 minutes at 4°C. We discarded the ethanol and allowed the pellets to dry. We resuspended the RNA in 50 μL of nuclease-free water and quantified them using Nanodrop and Qubit. We treated the samples with TURBO DNase (Invitrogen) to remove contaminating DNA. We cleaned up the RNA using 2× Ampure RNAclean beads (Beckman Coulter) and eluted RNA in 25 μL water. We generated cDNA libraries using the Illumina stranded mRNA ligation kit according to the manufacturer's protocol. Briefly, 100 ng of DNase-treated RNA was used to perform mRNA selection using oligo-dT linked beads. First-strand cDNA was then generated from polyadenylated RNA. Second-strand synthesis was performed using dUTP to achieve strand specificity. Illumina RNA index anchors and dual-index adaptors were then ligated. The libraries were amplified for 13 cycles and cleaned up with 1× volume of Ampure XP beads (Beckman Coulter). Fragment Analysis was performed to assess library quality and size distribution. Libraries were sequenced by Novogene (Durham, NC) on an Illumina Novaseq 6000 with 150 bp paired-end reads.

### Transcriptome assembly

We used data from all 3 tissue types (brain, leg muscle, and spinal cord) to assemble a de novo transcriptome for *S. parvus.* First, we concatenated all of the reads before randomly subsampling 20 million forward and 20 million reverse reads from each tissue type using seqtk (Li, 2023; https://github.com/lh3/seqtk). We concatenated all of these reads into a single forward and reverse dataset, composed of 60 million forward and 60 million reverse reads. We then assembled a de novo transcriptome using the Oyster River Protocol using a minimum filter of 1 Transcript Per Million (“TPM=1”) (MacManes 2018). This pipeline uses multiple assemblers and merges the assembled transcripts from each into a single assembly. We then used TransDecoder (https://github.com/TransDecoder/TransDecoder) to predict protein coding regions from the assembled transcriptome. We used “TrasDecoder.LongOrfs” to create a file of open reading frames and then used the Uniprot database (The UniProt Consortium 2019) and “blastp” (Camacho *et al*. 2009) as well as “hmmscan” (Zhang and Wood 2003) and the Pfam database (Mistry *et al*. 2021) to identify known peptides.

### Genome assembly

We converted the reads that the sequencer classified as “good” subreads from “.bam” format into fasta files using the samtools “fasta” command (Li *et al*. 2009). We then used the contig assembler wtdbg2 version 2.5 (Ruan and Li 2020) to create our initial assembly. Wtdbg2 uses a fuzzy de bruijn graph approach to combine reads into contigs. We set the kmer size to 21 (“-p 21”), subsampled half of our kmers (“-S 2”), removed reads smaller than 5,000 base pairs prior to assembling (“-L 5000”) per the software developers’ suggestion, and retained edges in the de bruijn graph that had low coverage (“–rescue-low-cov-edges”). We then used the function “wtpoa-cns” from wtdbg2 to error correct the initial assembly using aligned reads.

Following this, we did a second round of error correction using the program Racon version v1.4.19 (Vaser *et al*. 2017). To do this, we first aligned our PacBio reads back to the assembled genome using minimap2 version 2.10-r761 (Li 2018). We then used the reads and read alignments to error correct using Racon with default settings.

To try and rescue additional genetic content, we used our RNA sequencing (RNAseq) data to scaffold the genome. To do this, we used the subsampled RNAseq dataset used to assemble the transcriptome (i.e. 60 million forward and 60 million reverse reads across tissue types), aligned these reads to the draft genome using BWA (Li and Durbin 2009), and scaffolded with P_RNA_scaffolder (Zhu *et al*. 2018) using default settings.

We examined the genome quality in two main ways. First, we examined the presence of the genic content in our genome using miniBUSCO (Huang and Li, 2023) using the tetrapod database (tetrapoda_odb10; 2021-02-19). Our second method of genome examination was genome contiguity. We used the Assemblathon perl script (Bradnam *et al*. 2013) to calculate the overall numbers of scaffolds and contigs as well as contiguity metrics, such as N50, for both.

### Repeat modeling and masking

We modeled genomic repeats with Repeat Modeler2 (Flynn *et al*. 2020) using RepBase database 20170127. We identified the classified consensus output from Repeat Modeler2 using Transposon Classifier “RFSB” (Riehl *et al*. 2022). We used known repeats from vertebrates within a combined database of Dfam_3.1 and RepBase-20181026 (Bao *et al*. 2015) and our modeled repeats as a repeat library input to mask our genome using Repeat Masker version 4.1.2-p1 (Smit *et al*. 2013-2015).

### Genome annotation

We then annotated the genome using MAKER version 3.01.02 (Campbell *et al*. 2014). We used transcript evidence from our newly predicted coding regions of *S. parvus* to aid in annotation and used the Uniprot database from December 2020 (The UniProt Consortium 2019) as protein evidence to identify genes and gene products. Maker was run with repeat masking done by Repeat Masker version 4.1.0 (Smit *et al*. 2013-2015) and ab initio gene predictions done by SNAP. Maker was run with the “-fix_nucleotides” flag.

### Differential expression analysis

We used the RNAseq data to also conduct differential expression analysis to compare gene expression patterns of each tissue type. RNAseq data were aligned to our reference genome using STAR version 2.7 (Dobin *et al*. 2013) in the gene count mode (“–quantMode GeneCounts”), allowing for up to 20 multiple mappings (“–outFilterMultimapNmax 20”) and 10 mismatched bases (“–outFilterMismatchNmax 10”). We then quantified the expression of genes using the program “htseq-count” (Putri *et al*. 2022). We conducted the alignment and counts at the transcript level in order to observe the expression of different isoforms. Then, we normalized the gene counts with the trimmed mean of *M*-values (TMM) method and conducted DE analysis using edgeR to reveal the identity and number of transcripts that are unique to each tissue (Robinson *et al*. 2010). We used the default settings of the function filterByExpr() from edgeR to filter out low abundance genes, specifically genes with raw expression levels of less than 10 in more than 2 samples (2 is the smallest group size we had). These lenient criteria allow us to compile all possible genes that are differentially expressed in the neuromuscular system that could be relevant to the evolution of foot-flagging behavior. We feel these criteria are justified in this case, because evidence from mammals and birds suggests that genes that are expressed in a tissue-specific manner, especially those expressed at low levels, may evolve more rapidly (Axelson *et al*. 2008; Zhang and Yang 2015; Bentz *et al*. 2019; Hao *et al*. 2019). We determined differentially expressed genes (DEGs), or genes that are significantly different in expression levels between tissues, by producing a false discovery rate (FDR) for each gene based on the Benjamini–Hochberg method (Benjamini and Hochberg 1995), using average counts per million (CPM) and log2 fold change (Log2FC) values. Log2FC was calculated using the average CPM value of each tissue sample group using the glmQLfit() function. Thus, the fold change values that we report for DEGs represent the ratio between the average CPM of one tissue group (e.g. brain) and the average CPM of another (e.g. spinal cord).

We then conducted gene ontology (GO) analysis using the manual gene list search tool and the GO overrepresentation test (GO biological process complete, Fisher's exact test with FDR correction) on PANTHER to reveal the categorical function of DEGs in each tissue type and GO terms enriched in each tissue (Thomas *et al*. 2022). The PANTHER GO overrepresentation test requires an input list of gene names, and in our case, we input the DEGs for each tissue from pairwise comparisons. PANTHER also requires choosing a reference genome already loaded into the website to be used to match and identify orthologous genes from the input list. Out of the available genomes, *Xenopus tropicalis* was the most closely related species to *S. parvus* and the only frog genome. Thus, we chose it as our reference for the test. As a result, 86 out of 118 DEGs were uniquely mapped to the *X. tropicalis* genome for the spinal cord (differentially expressed from the brain) and 2,058 genes out of 3,220 DEGs and 2,480 out of 3,795 DEGs were uniquely mapped for leg muscle (differentially expressed from the brain and spinal cord, respectively).

### AR analyses

Previous evidence indicates the likely important role that AR plays in the evolution of foot-flagging behavior (Mangiamele *et al*. 2016; Anderson *et al*. 2021), therefore we were interested in identifying both gene expression of AR and how its sequence compares to related species. Due to a combination of AR's length, low expression levels in our samples, and the presence of nested genes within AR, the annotation of this gene was poor. We recovered only 5 exons, with no 5′ or 3′ regions. To get a better understanding of AR in *S. parvus* we then manually annotated this gene. We first extracted available AR protein sequences from frogs (genera *Bufo*, *Nanorana*, *Rana*, and *Xenopus*) as well as *Anolis carolinensis.* We then used blastx (Camacho *et al*. 2009) to map these proteins to the genomic scaffold containing AR. We extracted these matches and visualized individual exons using JBrowse2 (Buels *et al*. 2016), then extracted overlapping sequences from the *S. parvus* genome using bedtools “getfasta” (Quinlan and Hall 2010). We then aligned AR mRNAs using MAFFT version 7.515 (Katoh and Standley 2013) allowing for gaps (flag “–leavegappyregion”). We built a phylogenetic tree using IQ-TREE version 2.2.0.3 (Minh *et al*. 2020) with 1,000 ultra fast bootstraps (“-bb 1000”) and specifying *Anolis carolinensis* as the outgroup. We plotted the resulting tree using ggtree (Yu *et al*. 2017).

## Results and discussion

### Genome

In total, we had 312,472,731,406 bases of data across 32,110,985 reads of genomic data, with an average read length of 9,731 base pairs. Our final draft assembly was 3.98 Gbp in total length. This assembly was split into a total of 22,402 contigs, with a contig N50 of 611,229 base pairs. The assembly contained 22,068 scaffolds with a scaffold N50 of 627,225 base pairs. BUSCO analyses indicated that the genome has 91.4% of vertebrate orthologs in complete, single copies, 0.5% of orthologs were present in complete, duplicated copies, 3.5% were fragmented, and 4.6% were missing (N = 5,310 total orthologs).

### Repeats

We identified most of the *S. parvus* genome as repeat elements (62.46% repeats). This is similar to other work to date on amphibians, which indicates that they are rife with repeat elements spread throughout the genome (Sun *et al*. 2015; Rogers *et al*. 2018; Funk *et al*. 2021; Stuckert *et al*. 2021; Wang *et al*. 2021). Over a third of the genome are DNA transposons (41.87%), many of which were hob-Activator (12.93%) or Tc1-IS630-Pogo (10.93%). Retroelements make up a large portion of the genome as well (20.47%). The retroelements found within the *S. parvus* genome are dominated by LINEs which represent 7.41% of the genome and LTR elements that make up 13.0%. Like several other species of amphibian (e.g. the poison frogs *Oophaga pumilio* and *Ranitomeya imitator* and the caecilian *Ichthyophis bannanicus*), gypsy/DIRS1 make up a large portion of the genome (8.72%), potentially indicating a proliferation of these elements early in the history of amphibia. As we continue to produce newer amphibian genome assemblies of higher quality (e.g. contiguity, per-base-accuracy), we will be able to identify trends in repeat elements across this clade. Of particular interest are when these elements proliferated in genomes, and if particular families of repeat elements are more abundant in certain taxa within amphibia.

### AR sequence divergence

**Fig. 2.**
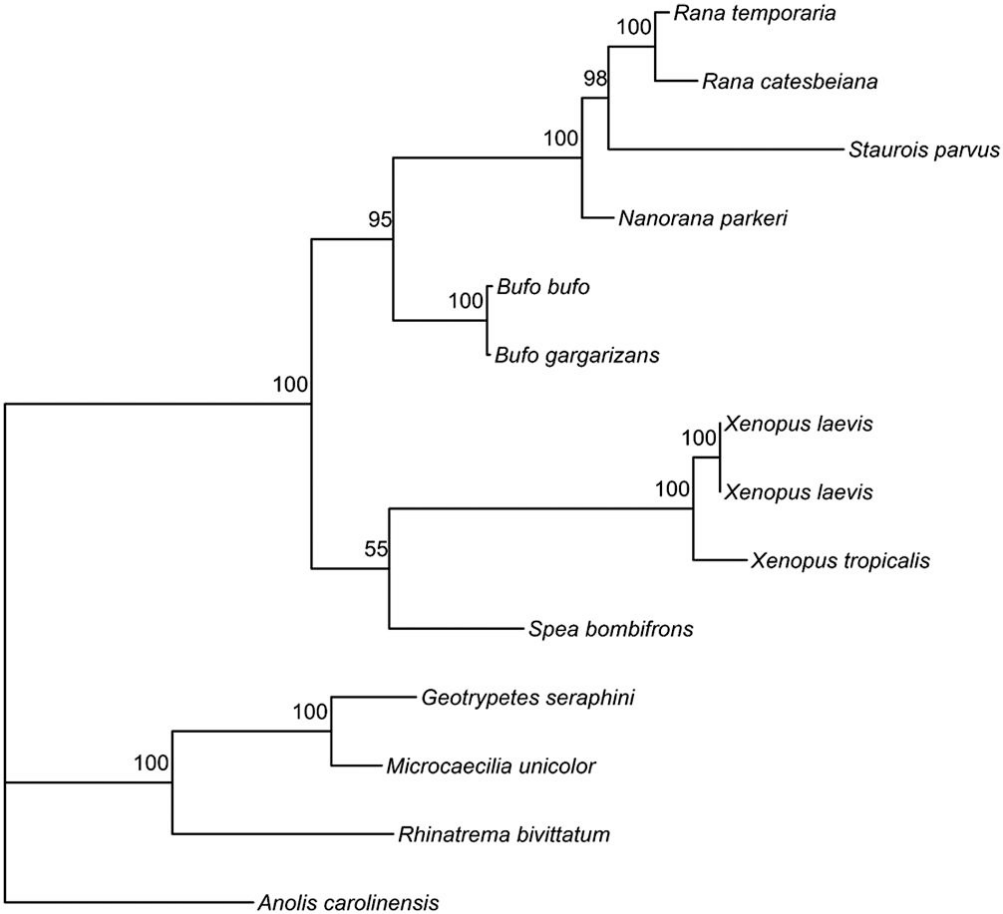
Consensus tree of the AR gene based on available cDNA sequences. Values at nodes indicate bootstrap support values.

Of particular interest is whether the AR gene in *S. parvus* has diverged from other frog species, given known species differences in AR expression that are associated with differences in sexual display behavior (Mangiamele *et al*. 2016; Anderson *et al*. 2021). We found that the *S. parvus* AR gene exhibits significant divergence from other ranids within the coding sequences of these species (Fig. 2; Supplementary Fig. 1). Further, we observed low gene expression of AR in our samples, likely precluding gene expression from being the only marker of behavioral evolution in foot-flagging frogs. Together, this indicates that protein evolution is also a plausible mechanism contributing to the emergence of foot-flagging behavior, as has been previously predicted (Mangiamele *et al*. 2016; Mangiamele and Fuxjager 2018). Further analysis of AR sequence, potentially in combination with full-length isoform sequencing, will be particularly informative in this system.

### RNAseq and differential expression

On average, each RNAseq readset contained 89,721,414 reads, with an 81.03% (±4.44% SD) mapping to the genome. We received a total of 240 GB of data across 11 samples that show similar gene expression patterns.

**Fig. 3.**
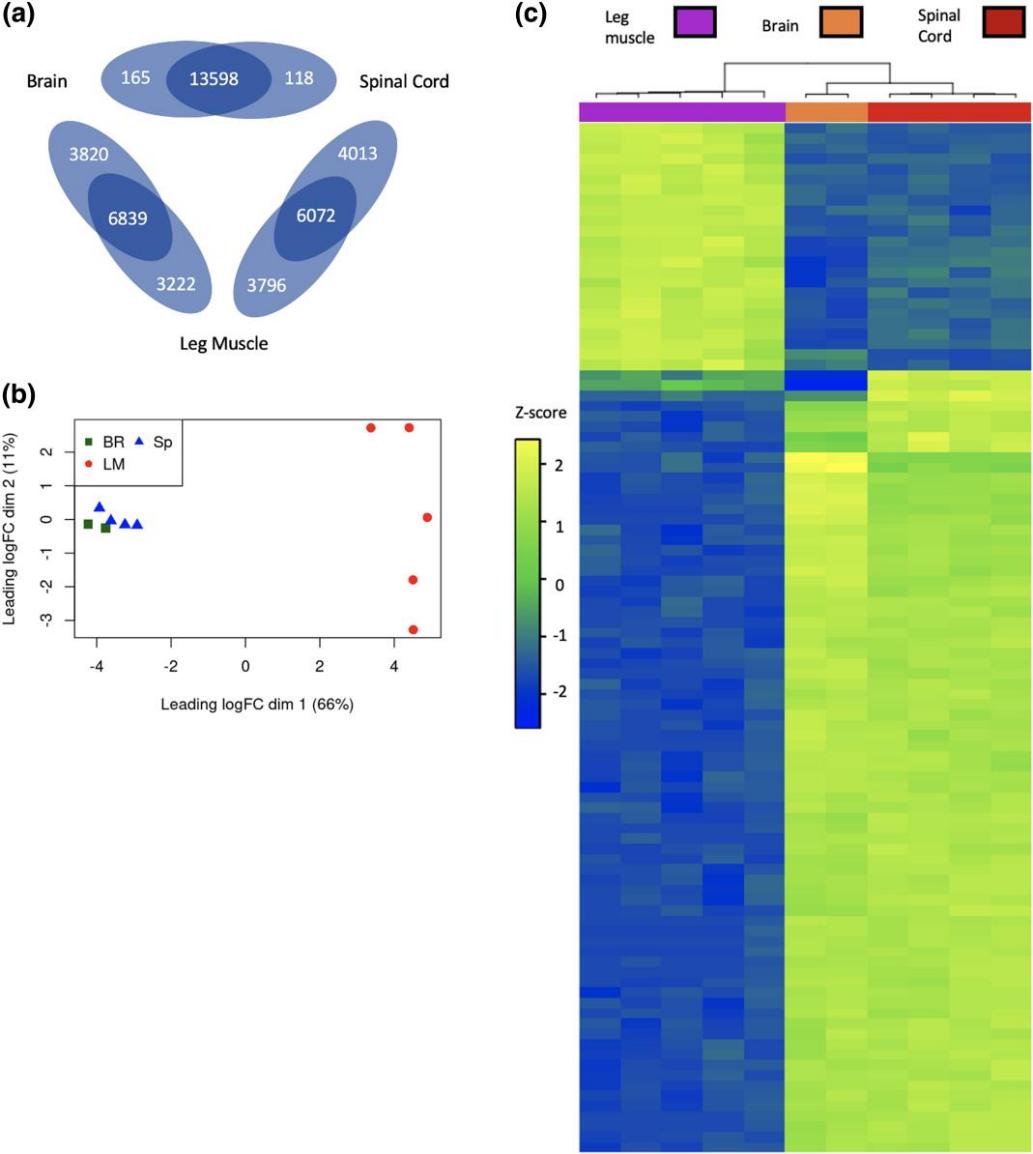
Distinctive patterns of gene expression associated with the nervous system and muscle tissue. a) Venn diagram showing the number of DEGs and genes shared in expression between tissue types in pairwise comparisons. b) Multidimensional scaling (MDS) plot comparing overall expression patterns between samples. BR, brain; LM, leg muscle; SP, spinal cord. c) Heat map of *z*-scores of the top 100 DEGs between tissue groups shows sample clustering based on tissue type. Yellow color indicates a higher expression of a gene in a sample compared with other samples while blue color indicates a lower expression. The dendrograms show the grouping of samples that show similar gene expression patterns. (For interpretation of the references to color in this figure legend, the reader is referred to the web version of this article).

The DE analysis revealed the number and identity of DEGs in each pairwise tissue comparison (Supplementary Table 1). All 3 comparisons had a total of close to 14,000 transcripts, where the comparison of the brain and spinal cord had the fewest DEGs (283 total) and the comparisons between leg muscle and the nervous system had thousands more DEGs (7,000–8,000) (Fig. 3a). Overall, the expression profiles of the brain and spinal cord are the closest, with leg muscle deviating from the other two (Fig. 3b). Among the top 100 DEGs (i.e. genes with the lowest FDR), we found the most divergent gene expression patterns between the nervous system and leg muscle tissues (Fig. 3c), presumably for supporting different functions within the neuromotor circuitry; for example, nervous system tissue must have the cellular machinery to send the electrical signals that coordinate movement, while muscle tissue must provide the contractile force to physically move limbs. Conversely, the brain and spinal cord have few DEGs and a more similar expression pattern, showing their similarity in structure and function, with slight differences in expression levels reflecting their specialized roles within the nervous system. However, this small group of genes could be useful in future studies that aim to help us understand the relative contributions of different neural pathways to the emergence of foot-flagging behavior. For example, from the 118 spinal cord DEGs, we might target genetic manipulations, such as loss of function, that we predict would primarily influence spinal control of foot-flagging behavior.

**Fig. 4.**
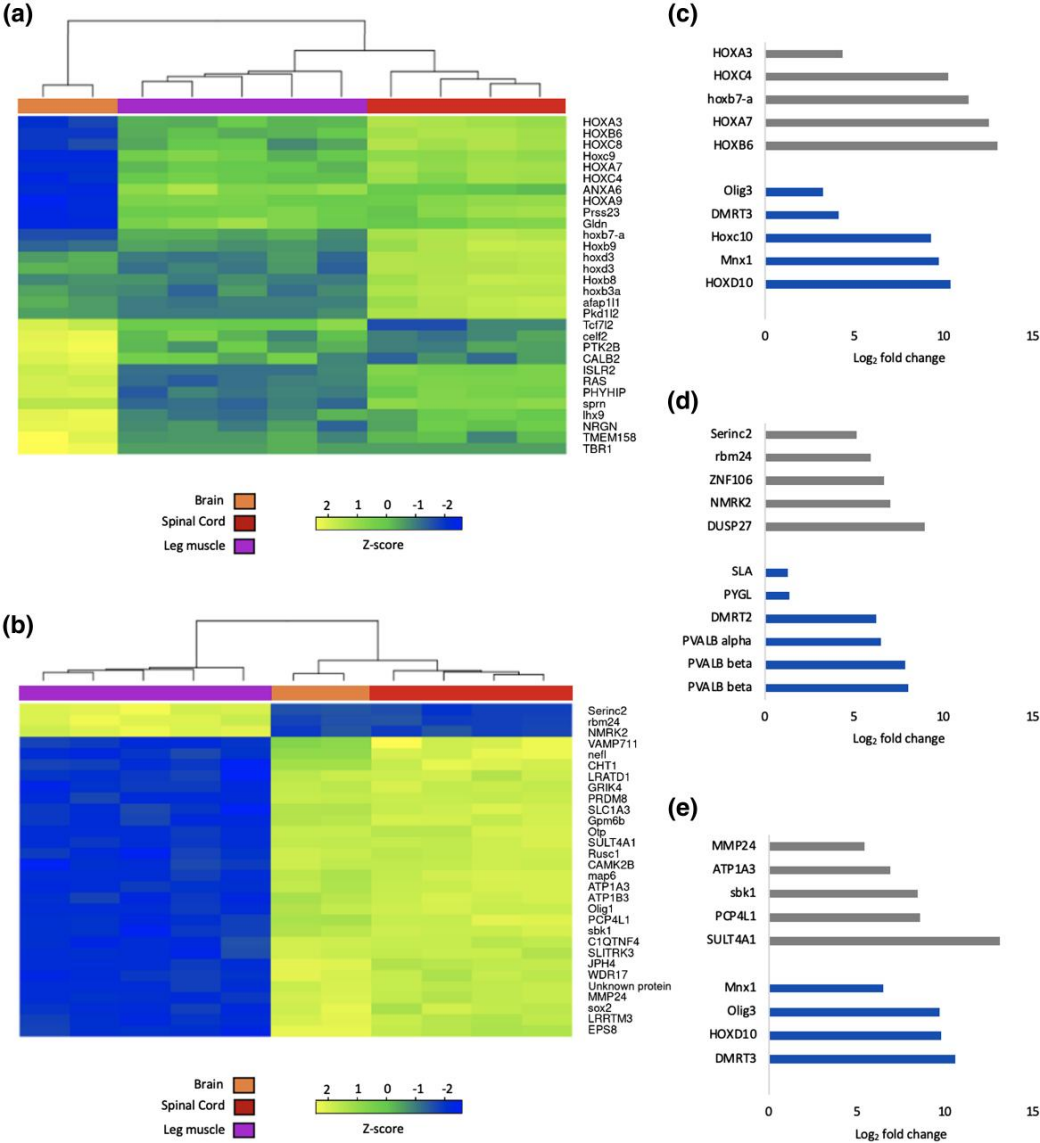
Pairwise comparisons reveal target genes that are most differentially expressed in neuromuscular tissues that control foot-flagging behavior. Heat maps depicting genes that are most differentially expressed between the (a) brain and spinal cord and (b) leg muscle and spinal cord. These heat maps highlight the top 30 genes within each comparison, while showing *z*-scores of these genes in every sample in our dataset. Tissues are shown on the *x*-axis and gene names are on the *y*-axis. Yellow color indicates a higher expression of a gene in a sample compared with other samples while blue color indicates a lower expression. The dendrograms show the grouping of samples showing similar gene expression patterns. Bar graphs compare absolute-value log2 fold changes of the top 5 genes (gray) and other genes of interest (blue) in the (c) spinal cord when compared with the brain, d) leg muscle when compared with the spinal cord, and (e) spinal cord when compared with leg muscle from the differential expression analyses. BR, brain; LM, leg muscle; SP, spinal cord. (For interpretation of the references to color in this figure legend, the reader is referred to the web version of this article).

Next, when we analyzed the top significant DEGs between tissue types in pairwise comparisons, several notable patterns emerged (Fig. 4). First, the top DEGs between the brain and spinal cord include a group of homeobox (HOX) genes that are highly expressed in the spinal cord but are much lower in the brain, with some showing a relatively higher expression in the leg muscle as well (Fig. 4a). Additionally, there is a different set of genes that are much more expressed in the brain compared with the spinal cord and leg muscle. When looking at the top DEGs between the spinal cord and leg muscle, there is a clear distinction between genes that are more expressed in one tissue than the other, with no gradient of expression levels between different tissues, meaning that these DEGs show robust differences in expression levels between the tissues (Fig. 4b).

### Gene ontology

The GO analysis helped categorize the DEGs, identify GO terms that were significantly enriched within each tissue, and pull out terms that could be related to the structure and function of hind limb motor circuitry in *S. parvus* (Supplementary Tables 2 and 3). Overall, the GO terms of interest for the spinal cord were related to spinal cord development and morphology, neuronal signaling, cell fate specification, and transcriptional processes. The GO terms of interest for leg muscle were related to muscle development, muscle contraction, metabolic processes, and motor actin dynamics. Then, the genes within these terms became of interest for future study (Supplementary Table 4).

Again, many of the key genes that emerged in our analysis were HOX genes (Fig. 4a and 4c), which are expressed primarily in the spinal cord. HOXC10 (Fig. 4c: abs(Log2FC) = 9.27) and HOXD10 (Fig. 4c: abs(Log2FC) = 10.4) in particular have previously been found to be important for lumbar spinal cord development and motor neuron organization and patterning in mice (Choe *et al*. 2006; Wu *et al*. 2008; Mallo *et al*. 2010). These two genes are also expressed into adulthood in *Xenopus* frogs (Kondo *et al*. 2017). Another HOX gene that was among the spinal cord DEGs was motor neuron and pancreas homeobox 1 (Mnx1, also known as homeobox HB9; Fig. 4c: abs(Log2FC) = 9.72). Knockouts and mutations of Mnx1 have led to impairments of motor axon projections, abnormal organization of spinal cells and motor neuron migration, and disorganized expression of interneuron and motor neuron marker genes in mice (Arber *et al*. 1999; Thaler *et al*. 1999). Future studies may manipulate the expression of these genes and observe their effects on the foot-flagging performance in *S. parvus* males (e.g. rate, speed, and path of the foot) or the morphology of the motor neurons that control hind limb movement in *S. parvus* and other foot-flagging frogs.

As prior studies have identified AR gene expression as a marker of divergence between foot-flagging and non–foot-flagging frog species, we first looked to see if this gene was included in our results. We did not find androgen signaling molecules among our significantly enriched GO terms, and AR was not differentially expressed across any of the tissues examined in this study. These results might be expected based on our prior qPCR study showing that *S. parvus* has similar AR expression levels in its hind limb musculature, spinal cord, and brain (Mangiamele *et al*. 2016). Yet, the AR itself is a transcription factor with many downstream genes whose expression depends not only on AR expression but also several cofactors (Schuppe and Fuxjager 2019). For example, SRC1 is a cofactor that upregulates AR activity. The SRC-like adaptor [SLA; Fig. 3d: abs(Log2FC) = 1.30], which contains the same SH domains as SRC1, was one of our DEGs; however, it is not clear whether it interacts with AR in the same way. In addition, previous work in birds has shown that androgen-regulated genes are highly expressed in muscle tissues that are used for the super-fast wing snap courtship displays of male golden-collared manakins (*Manacus vitellinus*) (Fuxjager *et al*. 2012, 2016). Although those genes were not in the top 100 most DEGs in our analysis, some of the androgen-regulated genes identified in Fuxjager *et al*. 2016 are also among *S. parvus*’ leg muscle DEGs. One such gene is parvalbumin (PVALB), which is involved in calcium signaling that allows for fast muscle relaxation [Fuxjager *et al*. 2012; Fig. 4d: PVALB alpha abs(Log2FC) = 6.47, PVALB beta type 1 abs(Log2FC) = 7.87, PVALB beta type 2 abs(Log2FC) = 8.03]. Another gene is glycogen phosphorylase (PYGL; Fig. 4d: abs(Log2FC) = 1.364), which is important for glucose synthesis from glycogen breakdown in muscle tissue (Newgard *et al*. 1989). These androgen-regulated genes, which appear to be highly expressed in the muscles of both manakins and foot-flagging frogs, may indicate that athletic courtship displays are driven by common downstream AR-regulated transcriptional changes in the neuromuscular system.

Doublesex and mab-3-related transcription factor (DMRT) genes have previously been studied for their role in sex differentiation and gonadal development, with diverse functions in other tissues (Hong *et al*. 2007; Picard *et al*. 2015). In *S. parvus*, we found that DMRT2 (Fig. 4d; abs(Log2FC) = 6.22) was differentially expressed in the leg muscle and DMRT3 (Fig. 4e; abs(Log2FC) = 10.6) in the spinal cord. DMRT2 has been shown to be important for skeletal muscle development and axial patterning in other vertebrate species (Seo *et al*. 2006; Seo 2007; Sato *et al*. 2010). DMRT3 has been studied for its role in the diversity of gait in horse species (Andersson *et al*. 2012). DMRT3 is expressed in the interneurons of networks important for limb locomotion and coordination, and inhibiting this gene resulted in abnormal gait and locomotor ability in mice (Perry *et al*. 2019; Vieillard *et al*. 2023). Based on this knowledge, perhaps both DMRT2 and DMRT3 have roles in the development of the neuromuscular system in *S. parvus* and their functions can be further studied by comparing the two sexes in *S. parvus* or seeing the effect that a loss of function of these genes could have on the development of the spinal motor circuit that contributes to foot flagging.

### Future directions

The foot-flagging frog, *S. parvus*, is an emerging model organism for studying the evolution of sexual display behaviors, especially those involving novel or impressive motor abilities. The de novo genome we present in this report will help us identify genes of interest that could be related to neuromotor system development or hind limb muscle performance, thus giving us direction for future experiments in manipulating such genes to understand their mechanistic role in foot flagging. We will also be able to use the genome as a resource for comparative genomics to understand in detail the genetic evolution between foot-flagging species and non–foot-flagging species, thereby identifying potential targets for selection on motor skills for sexual display. Future differential expression analyses will also help us identify patterns of gene expression associated with differences in motor behaviors and foot-flagging performance between sexes and between frog species (both foot flagging and non-foot flagging) and due to the effects of hormone treatments. The building of genetic knowledge on *S. parvus* is an exciting next step for further understanding the evolution of unique signaling behaviors.

## Supplementary Material

jkad193_Supplementary_Data

## Data Availability

All read data, de novo transcriptome assemblies, our de novo genome assembly, and our annotations are archived with the European Nucleotide Archive (accession number PRJEB40591; https://www.ebi.ac.uk/ena/browser/view/PRJEB40591). Code for assemblies, annotation, gene counts, and all subsequent analyses are available on GitHub (https://github.com/AdamStuckert/Footloose_genome). Raw RNASeq data from the *S. parvus* brain, spinal cord, and leg muscle tissue samples, along with a FASTA file of the assembled sequences, have been deposited in NCBI's Gene Expression Omnibus (Edgar *et al*. 2002) and are accessible through GEO Series accession number GSE232203 (https://www.ncbi.nlm.nih.gov/geo/query/acc.cgi?acc=GSE232203). Supplementary Video 1 contains a video of the foot-flagging display. Supplementary Table 1 contains raw results from the differential expression analyses. Supplementary Table 2 contains raw results from gene ontology overrepresentation analyses. Supplementary Table 3 contains select gene ontology terms of interest. Supplementary Table 4 contains genes of interest and their corresponding gene ontology terms. Supplementary Fig. 1 contains the consensus tree of the androgen receptor gene based on available predicted protein sequences. Note that the *S. parvus*-predicted protein is not full length. Supplemental material available at G3 online.
