# Supplementary material for: Genome assembly of the foot-flagging frog, Staurois parvus: a resource for understanding mechanisms of behavior
Source: G3 (Bethesda). 2023 Aug 25;13(10):jkad193. doi: 10.1093/g3journal/jkad193 (PMC10542557; doi:10.1093/g3journal/jkad193)
Supplement: jkad193_Supplementary_Data [file jkad193_supplementary_data.zip › G3-2023-404437_Table_S3.pdf]

Supplementary Table S3. Gene ontology (GO) biological process overrepresentation test results. GO terms of interest pulled from spinal cord and leg muscle DEGs.

**Spinal Cord**	FDR	**Leg Muscle**	FDR (vs brain)	FDR (vs spinal cord)
spinal cord motor neuron cell fate specification (GO:0021520)	2.09E-02	skeletal muscle tissue development (GO:0007519)	2.36E-02	8.55E-03
postsynaptic intermediate filament cytoskeleton organization (GO:0099185)	3.26E-02	branched-chain amino acid metabolic process (GO:0009081)	4.30E-02	3.38E-02
spinal cord motor neuron differentiation (GO:0021522)	4.61E-02	regulation of actin filament depolymerization (GO:0030834)	4.78E-04	2.56E-03
embryonic skeletal system morphogenesis (GO:0048704)	1.15E-02	striated muscle cell differentiation (GO:0051146)	1.57E-02	2.74E-04
embryonic skeletal system development (GO:0048706)	1.91E-02	ATP metabolic process (GO:0046034)	1.33E-04	2.73E-03
skeletal system morphogenesis (GO:0048705)	2.05E-02	actin filament capping (GO:0051693)	3.46E-04	1.66E-03
anterior/posterior pattern specification (GO:0009952)	2.12E-03	negative regulation of actin filament polymerization (GO:0030837)	1.23E-03	5.40E-03
pattern specification process (GO:0007389)	1.60E-02	cellular calcium ion homeostasis (GO:0006874)	3.50E-02	--
regulation of transcription by RNA polymerase II (GO:0006357)	3.47E-11	aerobic respiration (GO:0009060)	3.25E-02	4.12E-04
