# Supplementary material for: Genome assembly of the foot-flagging frog, Staurois parvus: a resource for understanding mechanisms of behavior
Source: G3 (Bethesda). 2023 Aug 25;13(10):jkad193. doi: 10.1093/g3journal/jkad193 (PMC10542557; doi:10.1093/g3journal/jkad193)
Supplement: jkad193_Supplementary_Data [file jkad193_supplementary_data.zip › G3-2023-404437_Table_S4.pdf]

Supplementary Table S4. GO terms of interest from the overrepresentation test and a manual search, and the DEGs (FDR<0.05) within them. Bold gene names are transcription factors.
* Terms that were manually searched for and not from the overrepresentation test

**Spinal Cord**	**GO term**	**Genes**	
	hormone binding*	GNRHR2	Gonadotropin-releasing hormone receptor 2 (pseudogene)
	developmental process involved in reproduction, sex differentiation*	**DMRT3**	Doublesex and mab-3 related transcription factor 3
		**KANK1**	KN motif and ankyrin repeat domains 1
	spinal cord motor neuron cell fate specification/differentiation	**Mnx1**	Motor neuron and pancreas homeobox 1
		**Olig3**	Oligodendrocyte transcription factor 3
	postsynaptic intermediate filament cytoskeleton organization	Nefl	Neurofilament light polypeptide
	(embryonic) skeletal system morphogenesis/development	**HOXD9**	Homeobox D9
		**HOXA3**	Homeobox A3
		mab21l2	Protein mab-21-like 2
		**HOXA9**	Homeobox A9
	anterior/posterior pattern specification	**HOXD9**	Homeobox D9
		**HOXA3**	Homeobox A3
		**HOXC6**	Homeobox C6
		**HOXA9**	Homeobox A9
		**HOXA7**	Homeobox A7
	regulation of transcription by RNA polymerase II (not in categories above)	**HOXC4**	Homeobox C4
		**HOXB9**	Homeobox B9
		**WT1**	Wilms tumor protein homolog
		**Pax8**	Paired box protein Pax-8
		**HOXC8**	Homeobox C8
		**HOXC10**	Homeobox C10
		**HOXB8**	Homeobox B8
		**HOXD3**	Homeobox D3
		**HOXC9**	Homeobox C9
		**HOXC5**	Homeobox C5
		**HOXC12**	Homeobox C12
		**DBX2**	Developing brain homeobox 2
		**Sox10**	Transcription factor Sox-10
		**HOXD10**	Homeobox D10

**Leg Muscle**	**GO term**	**Genes**	
	androgen/estrogen/progesterone biosynthesis*	DGAT1	Diacylglycerol O-acyltransferase 1
	developmental process involved in reproduction, sex differentiation*	**DMRT2**	Doublesex and mab-3 related transcription factor 2
		**KANK1**	KN motif and ankyrin repeat domains 1
		tdrp	Testis development-related protein
	skeletal muscle tissue development	BVES	Blood vessel epicardial substance
		MYBPC1	Myosin binding protein C1
		**Tcf21**	Transcription factor 21
		**Myf6**	Myogenic factor 6
		Klhl40	Kelch-like protein 40
		**Six1**	SIX homeobox 1

	**MYOG**	Myogenin
	**Myf5**	Myogenic factor
	PPARGC1A	Peroxisome proliferator-activated receptor gamma coactivator 1-alpha
	PLOD2	Procollagen-lysine 5-dioxygenase
branched-chain amino acid metabolic process	DBT	Dihydrolipoamide acetyltransferase component of pyruvate dehydrogenase complex
	HMGCL	3-hydroxy-3-methylglutarate-CoA lyase
	**IVD**	Butyryl-CoA dehydrogenase
	HIBADH	3-hydroxyisobutyrate dehydrogenase
	BCKDHA	2-oxoisovalerate dehydrogenase subunit alpha
	Mccc2	Methylcrotonoyl-CoA carboxylase 2
	Aldh6a1	Aldehyde dehydrogenase 6 family member A1
	BCAT1	Branched chain amino-acid transaminase 1, cytosolic
actin filament capping	Lmod2	Leiomodin 2
	Sptan1	Spectrin, alpha, non-erythrocytic 1
	SPTBN1	Spectrin beta chain
	Tmod4	Tropomodulin 4
	Lmod2	Coiled-coil-helix-coiled-coil-helix domain containing 3
	SPTB	Spectrin beta chain
	LMOD3	Leiomodin 3
	Tmod3	Tropomodulin 3
striated muscle cell differentiation	LRRC39	Leucine rich repeat containing 39
	Pgm5	Phosphoglucomutase 5
	TFPI2	Tissue factor pathway inhibitor
	pld3	5'-3' exonuclease PLD3
	MYBPC1	Myosin binding protein C1
	**MYOCD**	Myocardin
	BVES	Blood vessel epicardial substance
	CSRP3	Cysteine and glycine-rich protein 2
	myof	Myoferlin
	**MEF2C**	MADS box transcription enhancer factor 2, polypeptide C
	NEB	Nebulin
	Tmod4	Tropomodulin 4
	klhl40	Kelch-like protein 40
	popdc2	Popeye domain containing 2
	**six1**	SIX homeobox 1
	**myf5**	Myogenic factor
	TNNT2	Troponin T2, cardiac type
	Tmem182	Transmembrane protein 182
	PLOD2	Procollagen-lysine 5-dioxygenase
	**tbx1**	T-box transcription factor TBX1
ATP metabolic process	TPI1	Triosephosphate isomerase
	ak2	Adenylate kinase 2, mitochondrial
	pkm	Pyruvate kinase
	Pgpep1l	Pyroglutamyl-peptidase I-like
	ak2	Adenylate kinase 2, mitochondrial
	ATP5F1B	ATP synthase subunit beta
	aldob,ALDOB	Fructose-bisphosphate aldolase B
	GPI	Glucose-6-phosphate isomerase
	Atp5mf	ATP synthase membrane subunit f
	PFKM	ATP-dependent 6-phosphofructokinase
	Atp5f1a	ATPsynthase subunit alpha
	AK1	Adenylate kinase isoenzyme 1
	Atp5po	ATP synthase subunit O, mitochondrial
	PGAM2	Phosphoglycerate mutase
	ATP5PD	ATP synthase subunit d, mitochondrial
	ATP5F1C	ATP synthase subunit gamma
	ALDOA	Fructose-bisphosphate aldolase A
	ATP5PB	ATP synthase subunit b

	tmed1	Transmembrane emp24 domain-containing protein 1
	HK1	Hexokinase
	Atp5mc3	ATP synthase lipid-binding protein
cellular calcium ion homeostasis	STIM2	Stromal interaction molecule 2
	ATP13A1	ATPase type 13A1
	ATP13A4	Cation-transporting ATPase
	ATP2A1	Calcium-transporting ATPase
	Abcb7	Calcium uniporter protein
	STIM1	Stromal interaction molecule
	ccdc47	Coiled-coil domain containing protein 47
	CAV2	Caveolin
	dhrs7c	Dehydrogenase/reductase SDR family member 7C
	letm1	Mitochondrial proton/calcium exchanger protein
	MCU	Calcium uniporter protein
	RYR1	Ryanodine receptor 1
aerobic respiration	cs	Citrate synthase, mitochondrial
	NDUFB9	Complex I-B22
	Cox4i2	Cytochrome c oxidase subunit 4
	NDUFS1	NADH: ubiquinone oxidoreductase core subunit S1
	NDUFA8	NADH dehydrogenase [ubiquinone] 1 alpha subcomplex subunit 8
	COX7A2L	Cytochrome c oxidase subunit VIIa polypeptide 2-like
	MDH2	Malate dehydrogenase
	COX7C	Cytochrome c oxidase polypeptide VIIc
	plk1	Oxoglutarate dehydrogenase (succinyl-transferring)
	IDH3B	Isocitrate dehydrogenase [NAD] subunit, mitochondrial
	Aco2	Aconitate hydratase, mitochondrial
	sdhb	Succinate dehydrogenase [ubiquinone] iron-sulfur subunit, mitochondrial
	UQCR10	Ubiquinol-cytochrome c reductase, complex III subunit X
	UQCR11	Ubiquinol-cytochrome c reductase, complex III subunit XI
	DLST	Dihydrolipoamide S-succinyltransferase (E2 component of 2-oxo-glutarate complex)
	NDUFA10	NADH:ubiquinone oxidoreductase subunit A10
	ogdh	Oxoglutarate dehydrogenase (succinyl-transferring)
	IDH1	Isocitrate dehydrogenase [NADP]
	Atp5po	ATP synthase subunit O, mitochondrial
	Cox7a2	Cox7a2 protein
	NDUFS8	Complex I-23kD
	COX7B	Cytochrome c oxidase subunit VIIb
	Sdhaf2	Succinate dehydrogenase complex assembly factor 2
	UQCRQ	Complex III subunit 8
	COX7B	Ras and Rab interactor 2
	NDUFS2	Complex I-49kD
	IDH2	Isocitrate dehydrogenase
