# Supplementary figures and images for: Genome assembly of the foot-flagging frog, Staurois parvus: a resource for understanding mechanisms of behavior
Source: G3 (Bethesda). 2023 Aug 25;13(10):jkad193. doi: 10.1093/g3journal/jkad193 (PMC10542557; doi:10.1093/g3journal/jkad193)

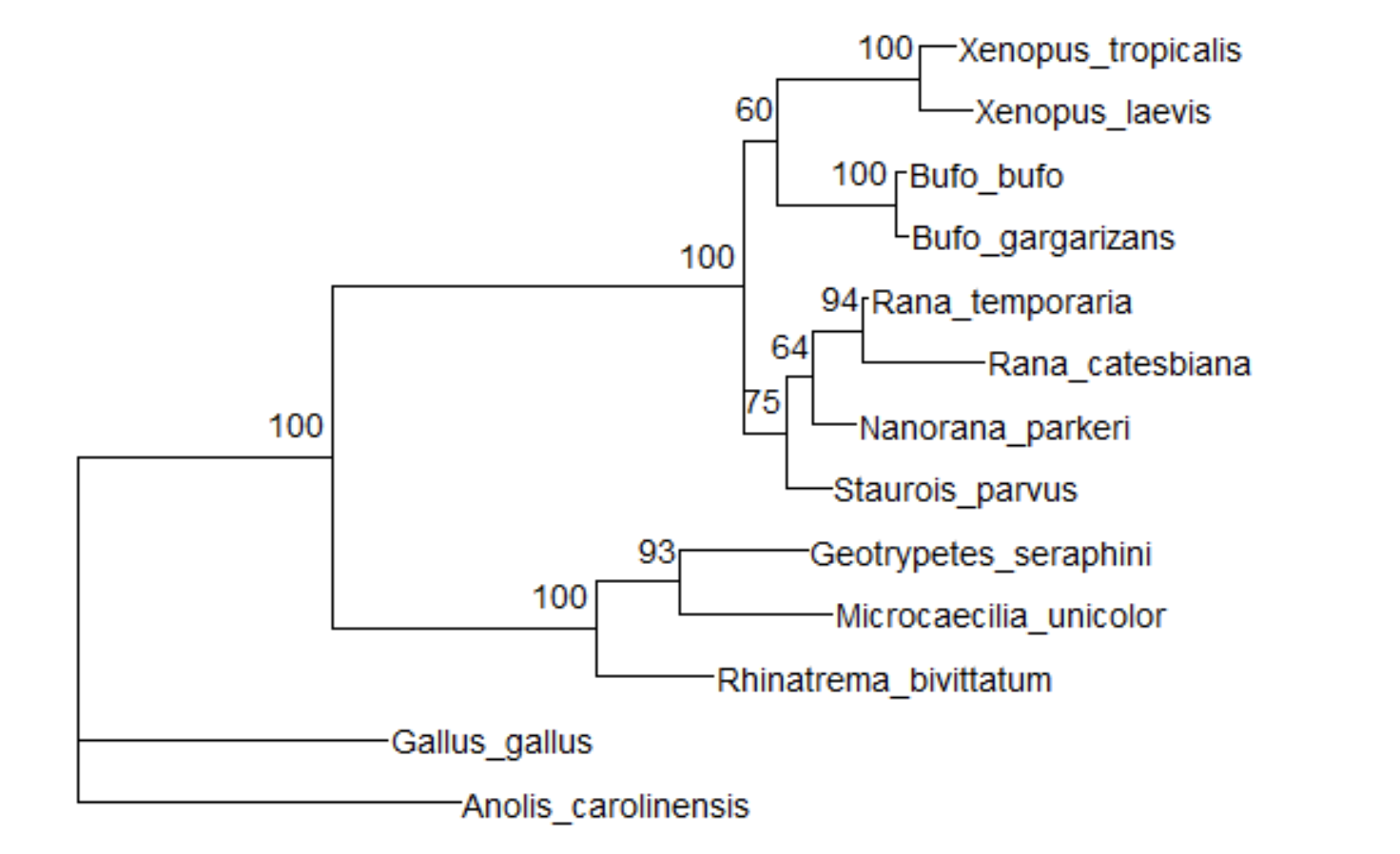

Supplement: jkad193_Supplementary_Data [file jkad193_supplementary_data.zip › G3-2023-404437_Figure_S1.png]
